# Supplementary figures and images for: Single-cell RNA-seq reveals disease-specific CD8+ T cell clonal expansion and a high frequency of transcriptionally distinct double-negative T cells in diabetic NOD mice
Source: PLoS One. 2025 Mar 19;20(3):e0317987. doi: 10.1371/journal.pone.0317987 (PMC11922263; doi:10.1371/journal.pone.0317987)

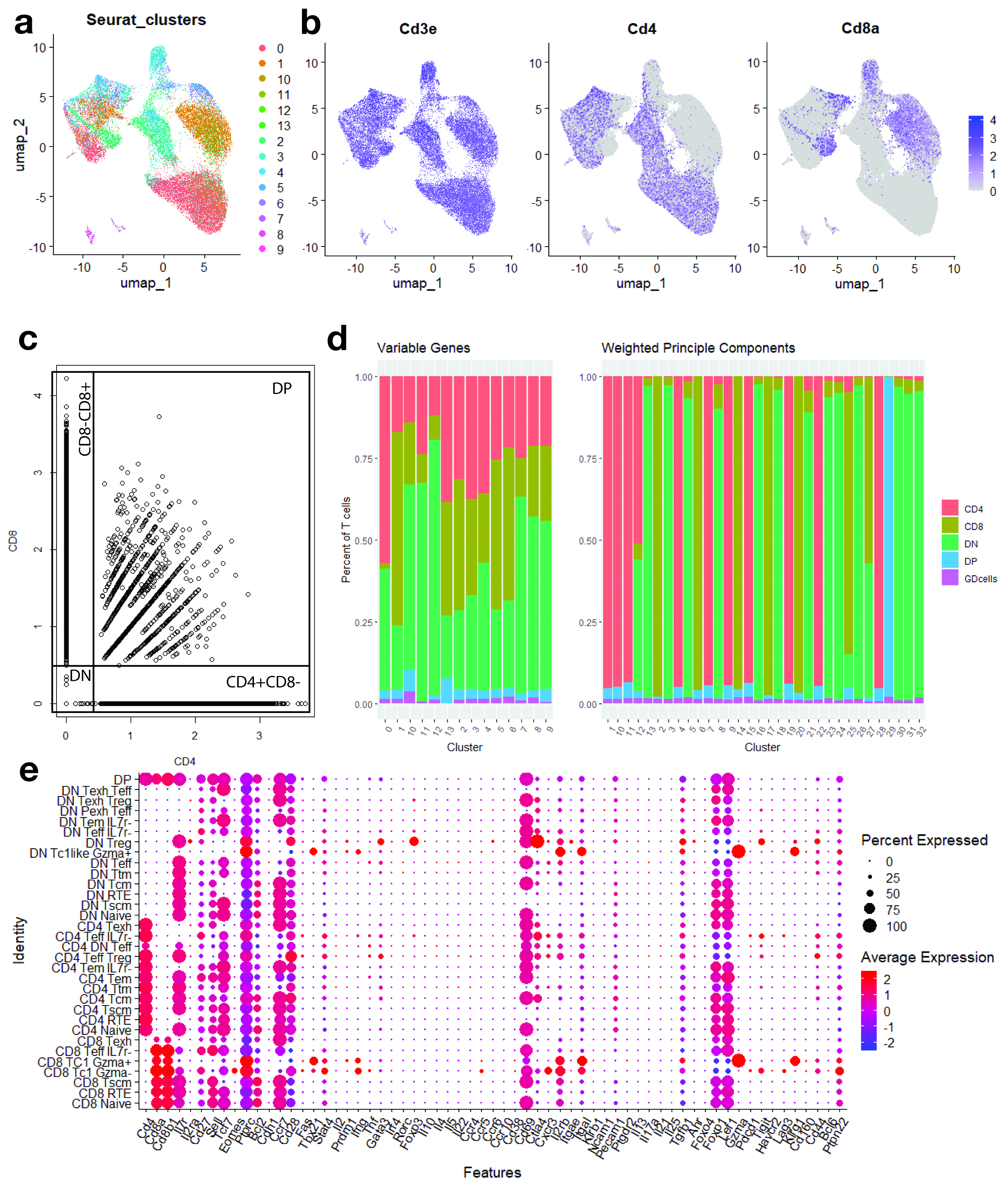

Supplement: S1 Fig — (a) Clustering and UMAP of high quality T cells with principal components generated from top 2000 most variably expressed genes. (b) Expression of CD3, CD4, and CD8 across cells plotted on UMAP from variably expressed gene principal components. (c) Expression of CD4 and CD8a in each cell. Gating on normalized expression was performed at a positive value over 0.05 for CD4 or CD8a. (d) Gated cells represented as a proportion of CD3 + T cells in either clusters generated from variably expressed genes alone or variably expressed genes with T cell marker weighted principal components. (e) Dotmap expression of T cell markers where the size of each dot represents the number of positively expressing cells and color is a continuous variable of average normalized expression across all cells. (TIF) [file pone.0317987.s001.tif]

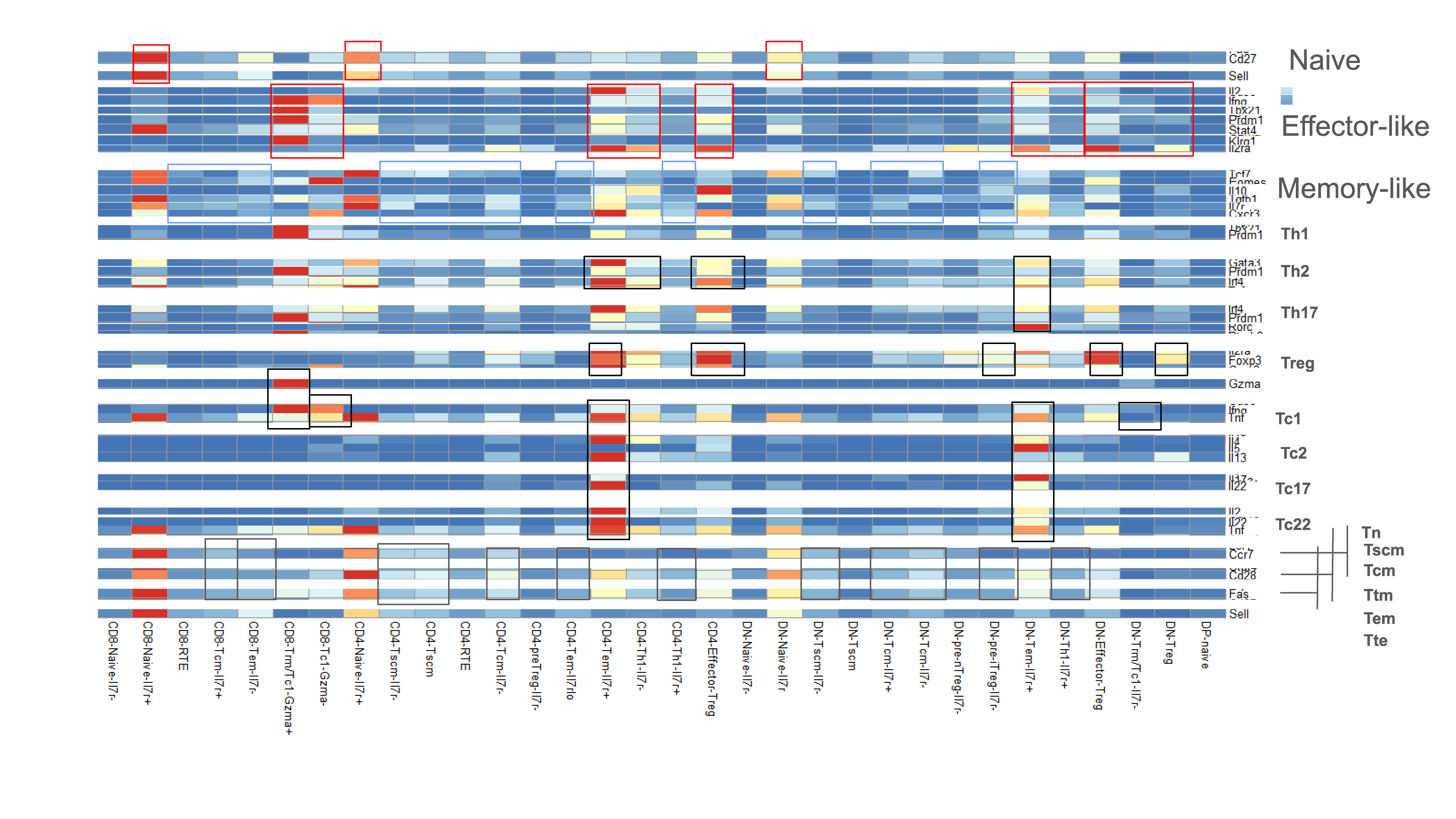

Supplement: S2 Fig — (TIF) [file pone.0317987.s002.tif]

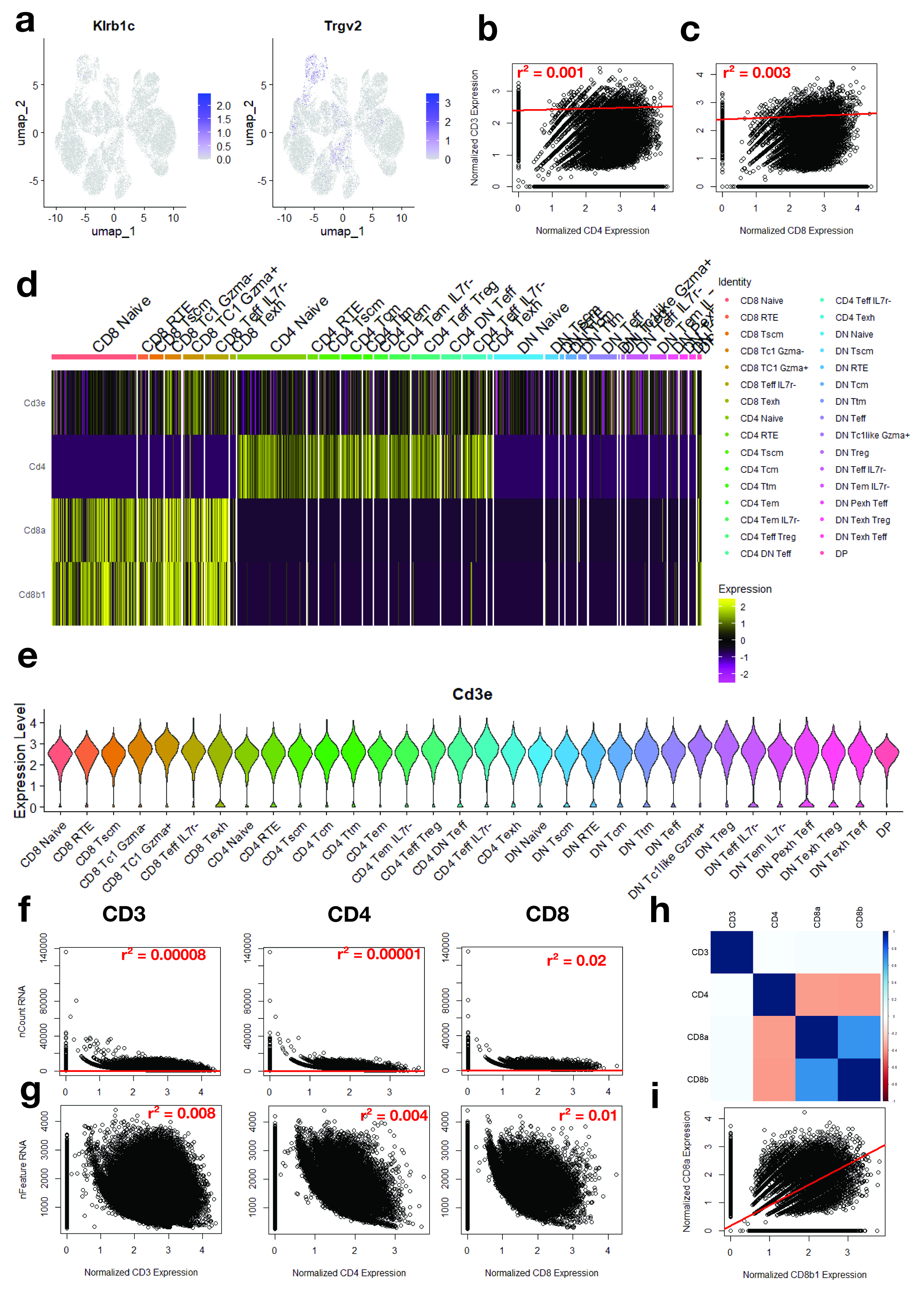

Supplement: S3 Fig — (a) Expression of Klrb1c (NKT cell marker) and Trgv2 (gamma delta T cell marker) plotted against the UMAP coordinate of each cell. Correlation of CD3e expression to (a) CD4 and (b) CD8a with red line representing a linear model of values. (d) Heatmap and (e) violin plot showing expression of CD3e across clusters demonstrates equivalent expression of CD3e. Expression of CD3e, CD4, and CD8a plotted against (f) nCount and (g) nFeatures for each cell. Red line represents a linear model for x and y values. (h) Correlation matrix plotted as a heatmap for expression of each marker with the linear correlation of (i) Cd8a and Cd8b demonstrating the heavy linkage between the two gene’s expression. (TIF) [file pone.0317987.s003.tif]

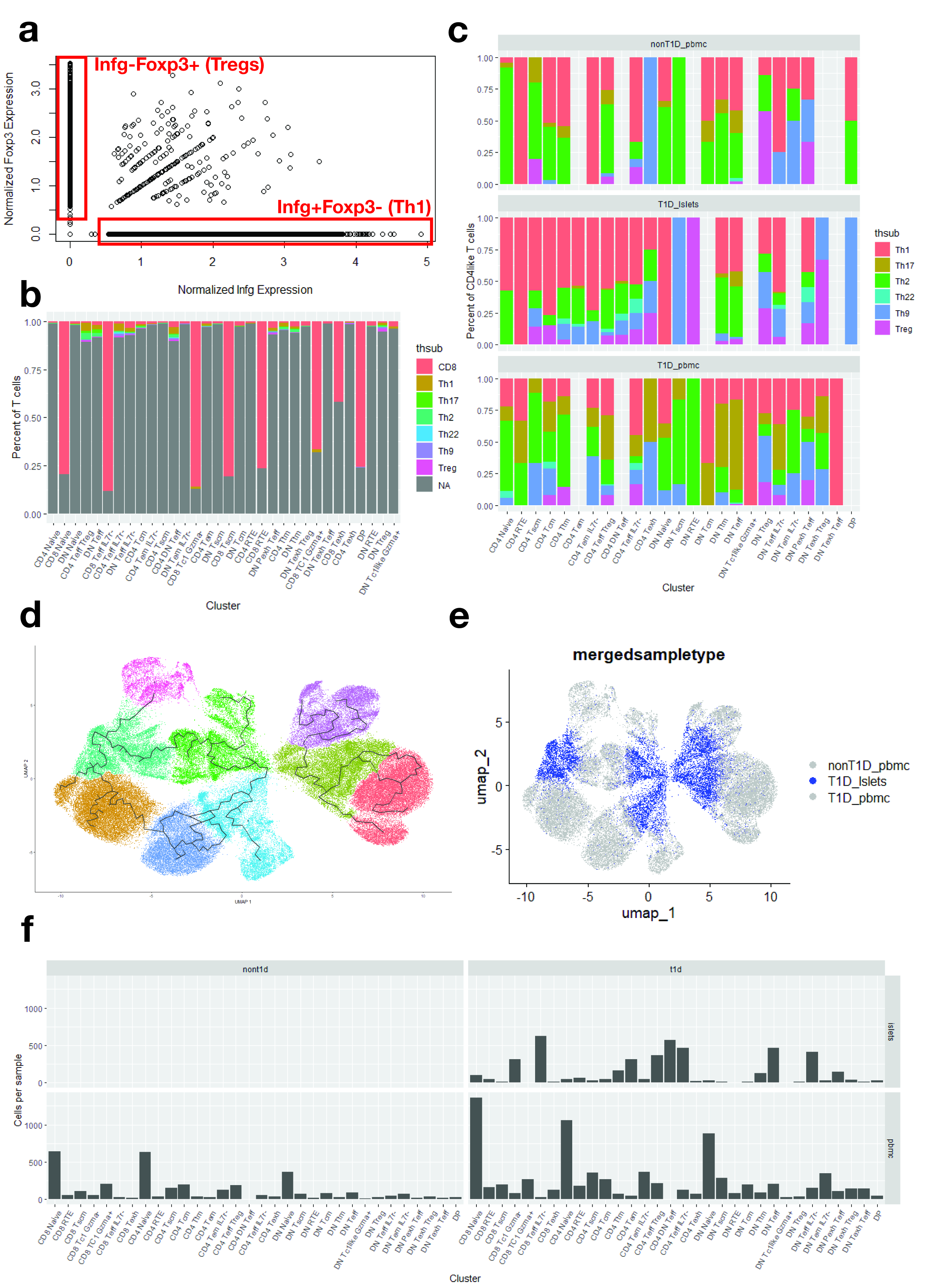

Supplement: S4 Fig — (a) Gating strategy example for identification of CD4 + T cell like subsets. T cell subsets were predicted as a lack of CD8 expression and concurrent expression of the following markers: Th1(Infg and TnfA), Th2 (IL-4 without IL-10 expression), Th17 (IL-17), Th22 (IL-22 and TNFA), Tfh (IL-4 and IL-10), Th9 (IL-10 without Ifng or TNFA expression), and Treg (Foxp3 and IL-10). T cells without those combination of marker expression were labeled NA. (b) Proportion of T cell subsets across clusters (average of 20% not NA found in each cluster). (c) Proportion of CD4 + T cell across CD4 and DN clusters separated by tissue. (d) Monocle clustering and trajectory in Seurat UMAP space. (e) Localization of islet T cells in UMAP space. (f) Average cells per sample per cluster faceted by disease condition and tissue source. (TIF) [file pone.0317987.s004.tif]

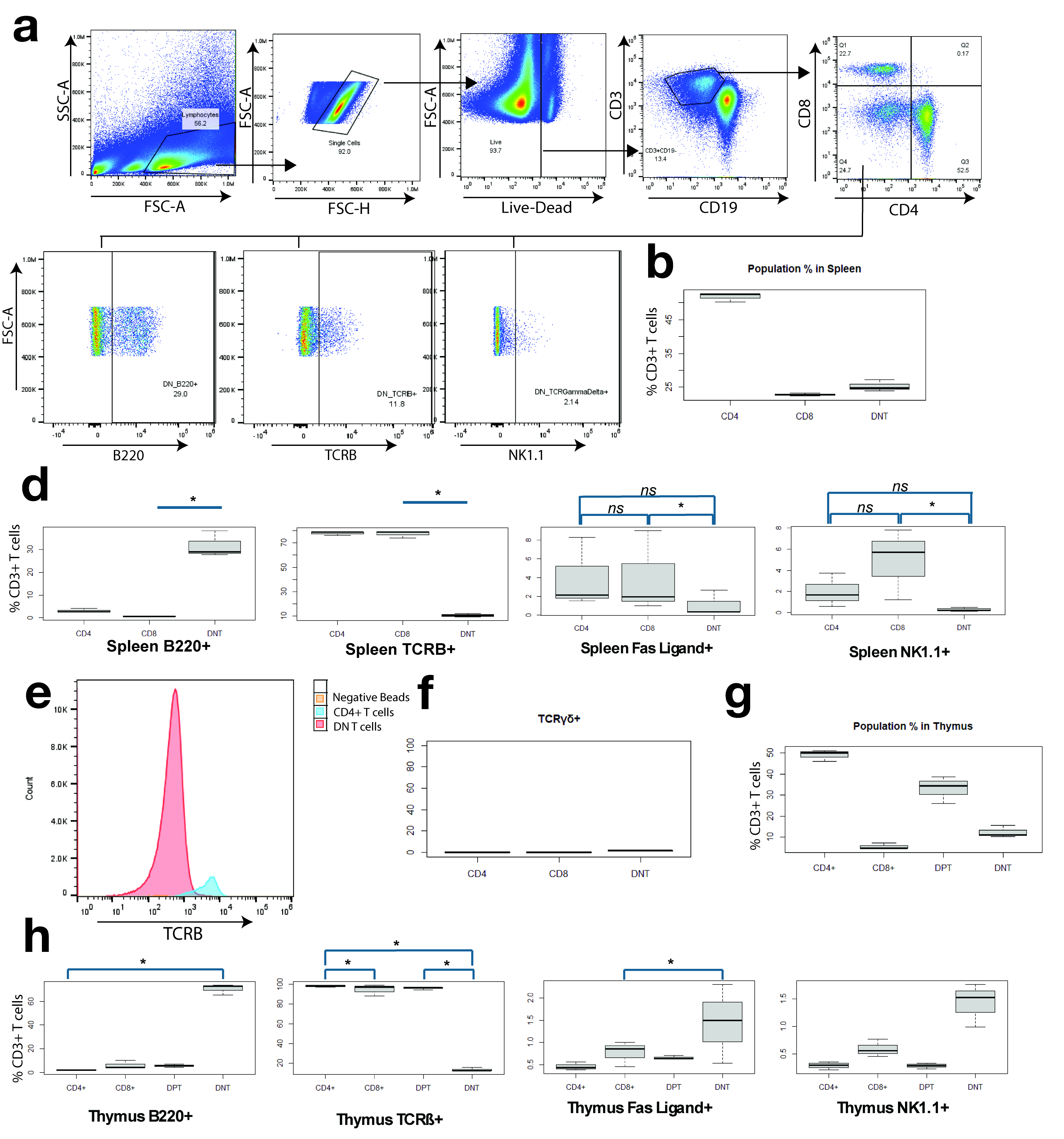

Supplement: S5 Fig — (a) Splenocytes were pooled from 24-week-old diabetic and non-diabetic NOD mice and then stained for T cell specific markers. (b) Boxplots show populations of CD3 + splenocytes of DN, CD4 + and CD8 + T cells. (c) Histogram of TCRB MFI shows lower protein abundance of TCR on the surface of DN T cells than CD4 + T cells. (d) Splenocytes largely are not expressers of gamma delta TCR. (e) Overall and (f) marker populations of CD3 + T cells in thymocytes. (TIF) [file pone.0317987.s005.tif]

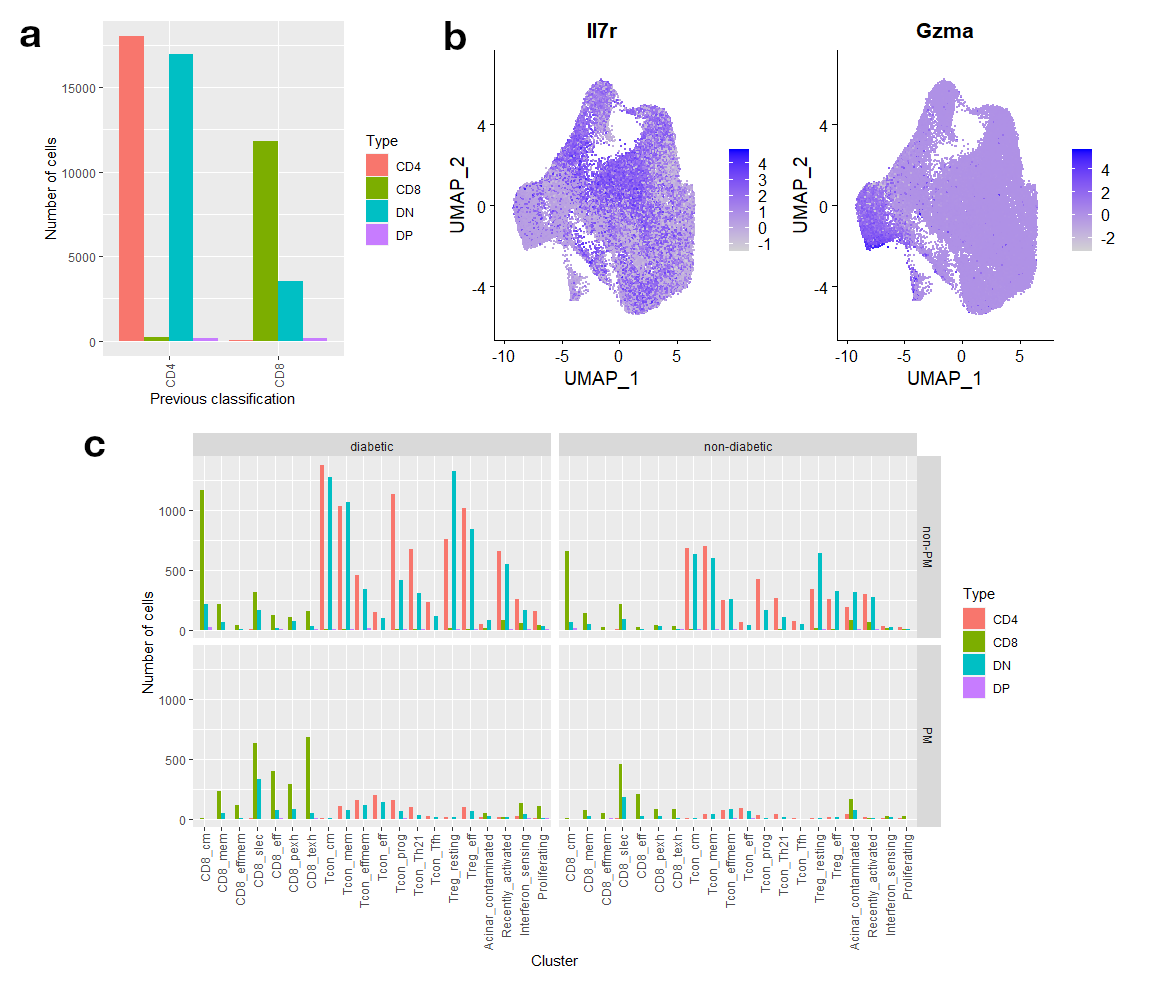

Supplement: S6 Fig — (a) Misclassification of DN T cells gated as described within this studies dataset. (b) Expression of markers IL7R and GzmA plotted on Collier et al., UMAP coordinates. (c) Average number of cells per sample per pre-annotated cluster faceted by disease and infiltration status and colored by the correct T cell compartment. (TIF) [file pone.0317987.s006.tif]

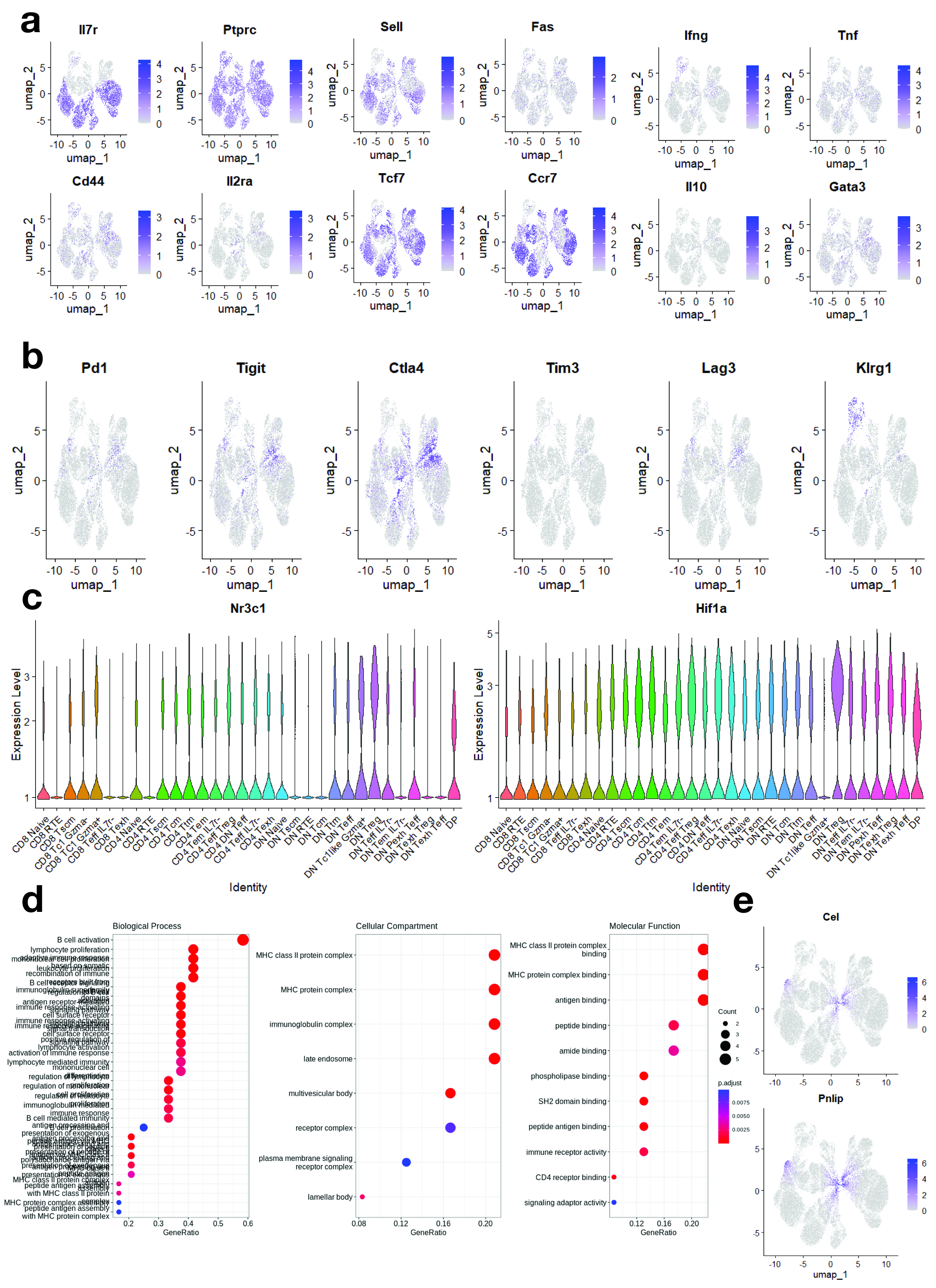

Supplement: S7 Fig — (a) Expression of commonly used markers in flow cytometric analysis of T cell populations. (b) Expression of exhaustion markers in T cells plotted in UMAP space. (c) Violin plot showing expression of Nr3c1 and Hif1a across clusters. (d) GSEA of grey module (MEGrey) using GO terms for biological process, cellular compartment, and molecular function. (e) Expression of Cel and PNLIPR across cells plotted in UMAP space. (TIF) [file pone.0317987.s007.tif]

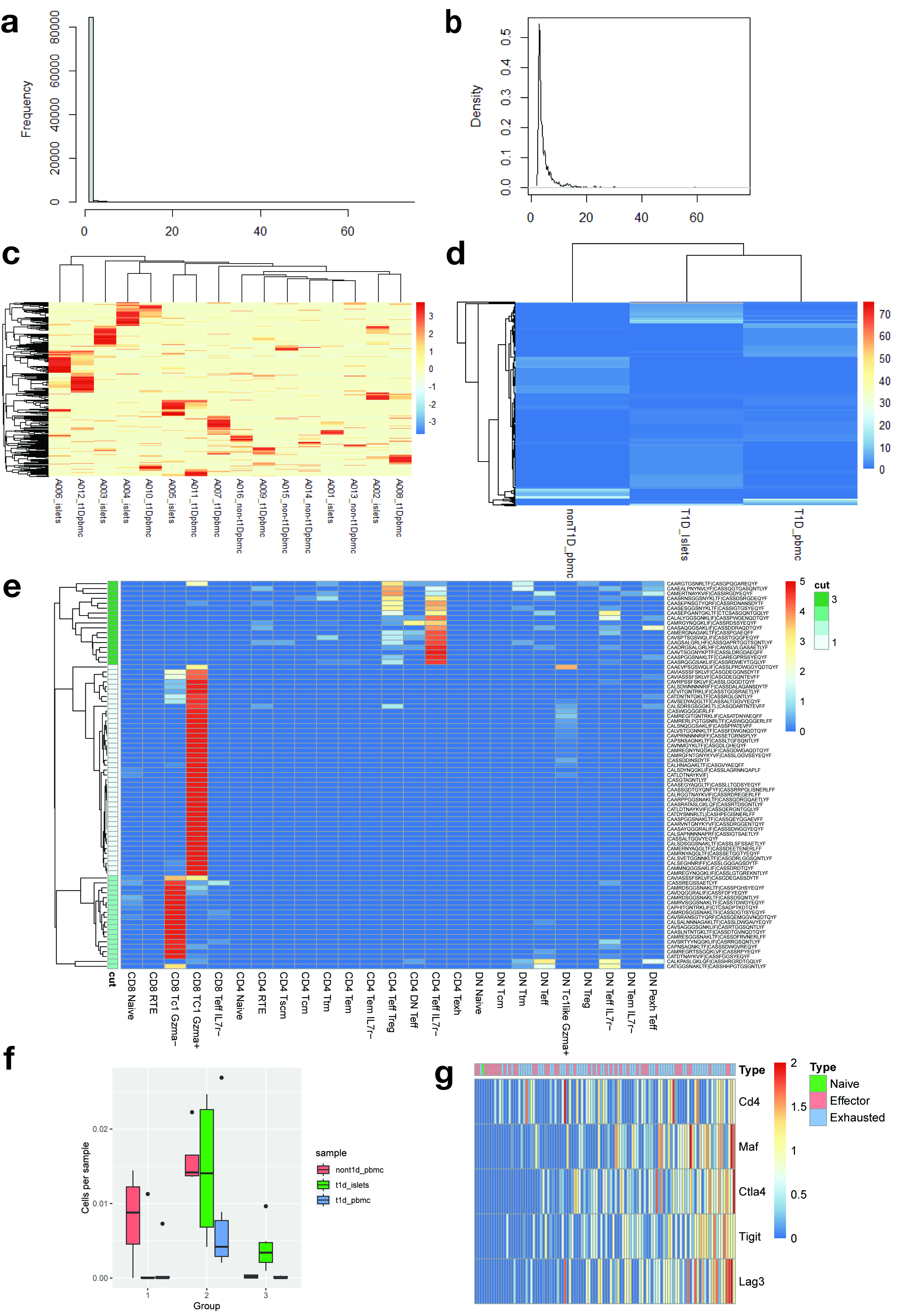

Supplement: S8 Fig — (a) Histogram showing the number frequency of TCR sequences on the y axis with counted cells > 0 on the x axis. (b) Density plot showing the relative frequency of single TCR sequences found in greater than 2 cells. Heatmaps showing the relative number of T cells for each TCR by row over the (c) sample and (d) tissue in columns. (e) Heatmap showing the frequency of TCR clones that have more than 10 cells in the entire dataset. Color bars on the left represent the group identity of the TCR clone that is used for differential expression analysis in Supplementary File 14. (f) The number of cells per sample is plotted for all cells belonging to each group of TCR sequences. The color represents which sample type and tissue the number of cells are calculated from. (g) Heatmap showing the relative expression of exhaustion marker genes in group 3 CD4 + and DN T cells. The color bars labeled type show the category of each cell’s cluster identity. Exhausted cell clusters are more associated with a higher level of expression of exhaustion markers. (TIF) [file pone.0317987.s008.tif]

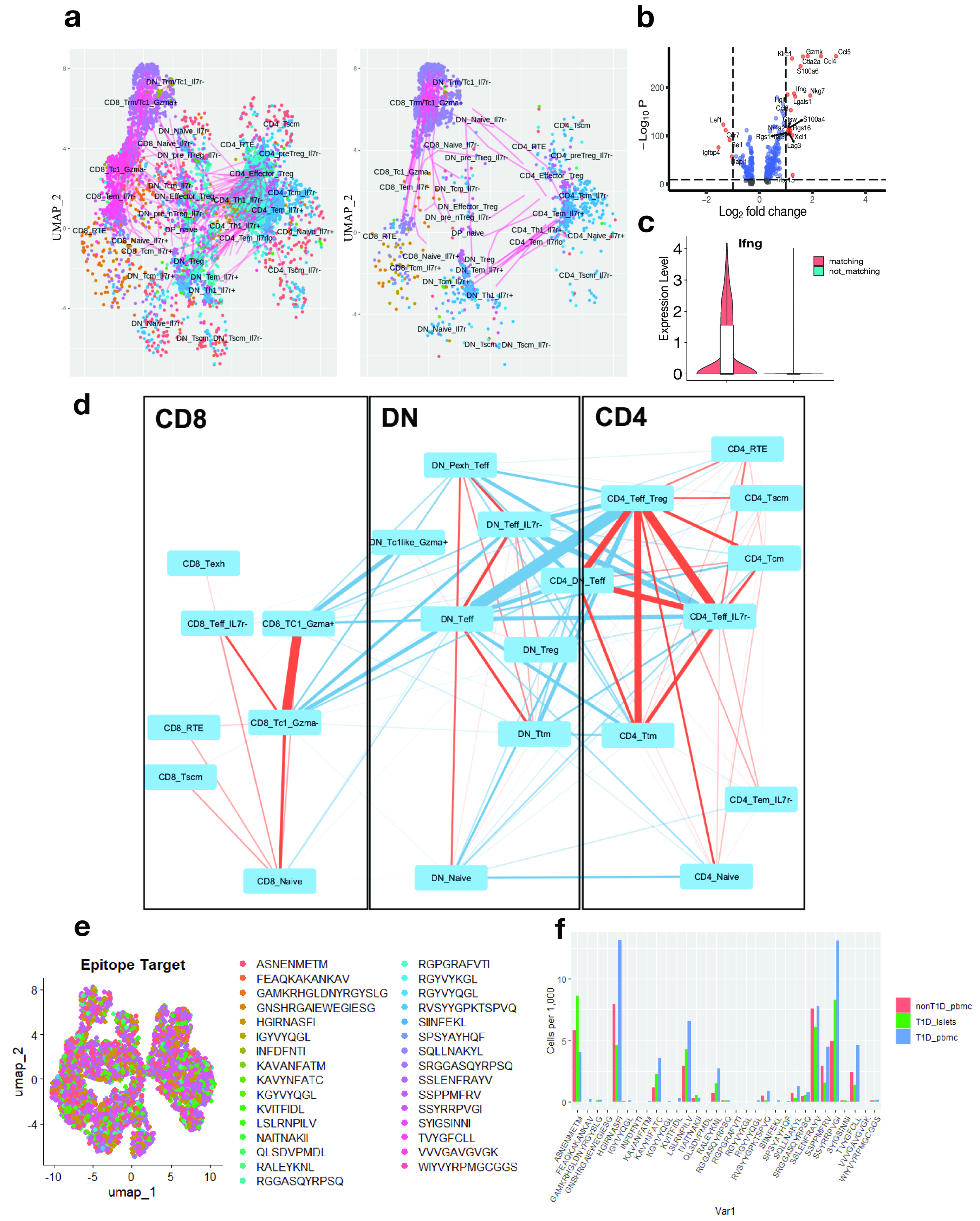

Supplement: S9 Fig — (a) Per TCR monocle trajectories plotted for each cell in the diabetic (left) and non-diabetic (right) mice. Each line represents the trajectory of one TCR clone across UMAP positions. (b) Volcano plot showing differential expression of genes in the infiltrating vs non-infiltrating T cells in diabetic mice with positive log fold change in the infiltrating T cells. Significant log fold change values > 1 and adjusted P-values < 0.01 are colored red and top 25 significant genes are labeled. (c) Violin and box plot showing average expression of Ifng between tissue matched (infiltrating) and non-matched T cells. (d) Likelihood estimation for bootstrapped pair transitions within clones. Each clone was randomly sampled for pairs of cells 100 times and the likelihood of each transition was calculated based on the proportion of two different cell types being chosen. The results were plotted as a graph with the nodes representing each cell cluster. The thickness of each bar represents the average percent likelihood of a transition occurring across clones with more than 3 cells. Red colored lines represent transitions occurring within the same compartment (i.e., CD4 to CD4) while blue colored lines represent transitions occurring between compartments (i.e., DN to CD4). The direction of transition cannot be inferred however the nodes are organized from bottom to top in order of ascending T cell maturation. (e) Epitope targets were predicted from the VDJdb database and cells with exact matches were plotted on the cell UMAP and colored by target epitope. (f) Normalized cell numbers were plotted based on targeted epitope by tissue. (TIF) [file pone.0317987.s009.tif]

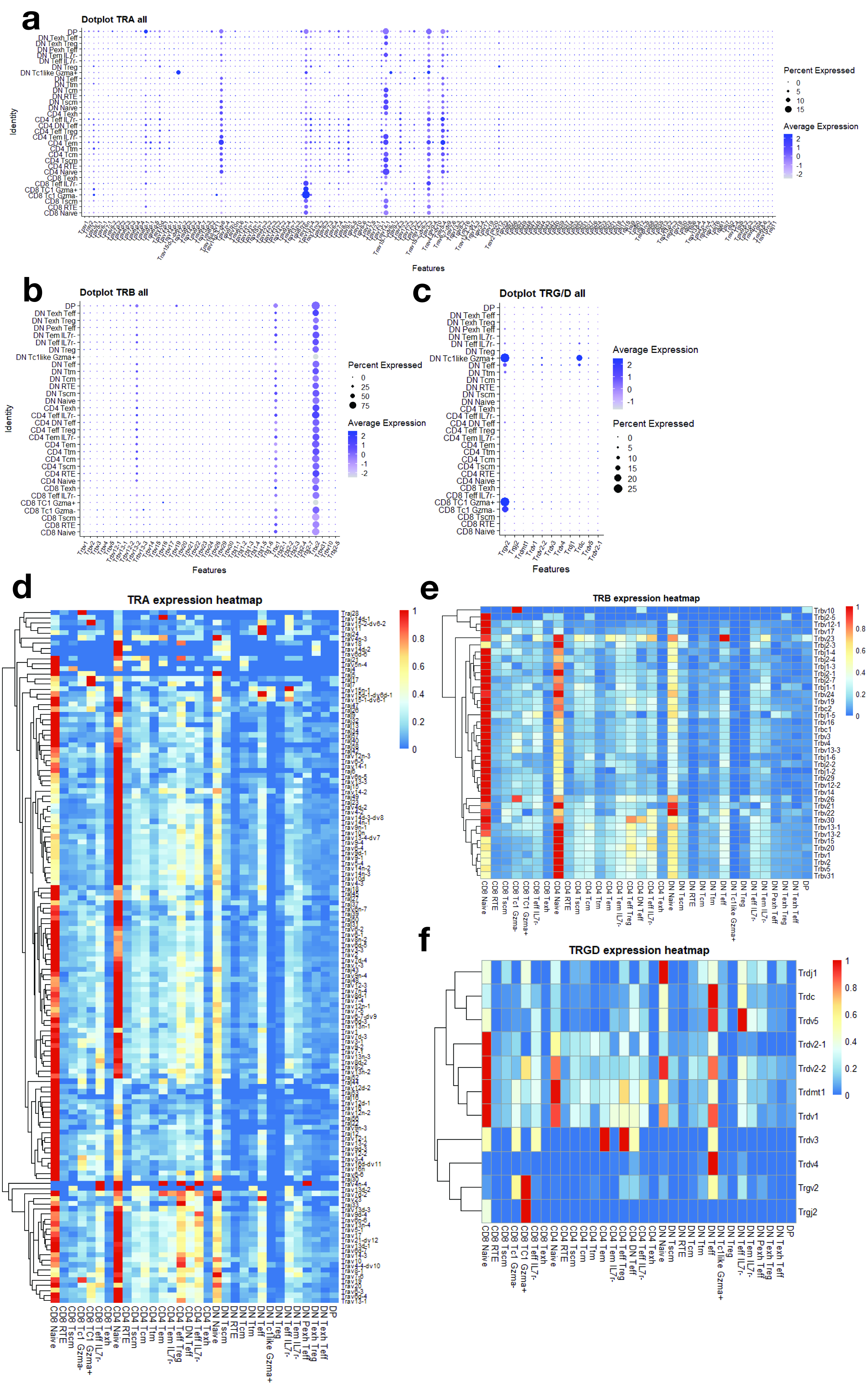

Supplement: S10 Fig — Dot plot showing cell percentage and average expression in each cluster for tcr alpha (a), beta (b), and gamma/delta (c). (d-f) Psdobulk expression plots showing average expression of tcr gene expression by cluster. (TIF) [file pone.0317987.s010.tif]
