# Supplementary material for: Single-cell RNA-seq reveals disease-specific CD8+ T cell clonal expansion and a high frequency of transcriptionally distinct double-negative T cells in diabetic NOD mice
Source: PLoS One. 2025 Mar 19;20(3):e0317987. doi: 10.1371/journal.pone.0317987 (PMC11922263; doi:10.1371/journal.pone.0317987)
Supplement: Attachment — Submitted filename: Response_to_reviewers_PLOSONE_MR_11_26_24.docx [file pone.0317987.s012.docx]

Dear editor and reviewers,

We would like to thank the editor and the reviewer for the opportunity to revise our manuscript to fall in line with the requirements of PlosONE. We have conducted a review of the suggested changes and completed additional analysis to satisfy all comments. We have also changed the main manuscript text, supplementary figures, and supplementary files. We will copy all changed text and figures within this response letter for the convenience of the editor and reviewers and will be happy to provide any additional information that is required for publication. Below we address each reviewer's concern with the initial comment in **bolded text**, the response in blue and any changed text from the manuscript in red text.

**Journal Requirements:**

**1. When submitting your revision, we need you to address these additional requirements.**

**Please ensure that your manuscript meets PLOS ONE's style requirements, including those for file naming. The PLOS ONE style templates can be found at**
[**https://journals.plos.org/plosone/s/file?id=wjVg/PLOSOne_formatting_sample_main_body.pdf**](https://urldefense.com/v3/__https://journals.plos.org/plosone/s/file?id=wjVg*PLOSOne_formatting_sample_main_body.pdf__;Lw!!DZ3fjg!-Wser9g61HL-T-rlfhdktLIyStMmWJl23m2BaQ2rbYwpCNtklsOcY--FUd6CWnjxZCuGiq1qPx2pAILxwg$) **and**
[**https://journals.plos.org/plosone/s/file?id=ba62/PLOSOne_formatting_sample_title_authors_affiliations.pdf**](https://urldefense.com/v3/__https://journals.plos.org/plosone/s/file?id=ba62*PLOSOne_formatting_sample_title_authors_affiliations.pdf__;Lw!!DZ3fjg!-Wser9g61HL-T-rlfhdktLIyStMmWJl23m2BaQ2rbYwpCNtklsOcY--FUd6CWnjxZCuGiq1qPx3SjS4dsg$)

**Author’s response:** We have made the necessary changes to comply with the PLOS ONE style.

**2. To comply with PLOS ONE submissions requirements, in your Methods section, please provide additional information regarding the experiments involving animals and ensure you have included details on (a) methods of sacrifice, (b) methods of anesthesia and/or analgesia, and (c) efforts to alleviate suffering.**

**Authors’ response:** We have added to the methodology section. Please see page line 484-487

“Briefly, approximately 200 µL of blood was collected through the lateral tail every two weeks. At the time of organ collection, mice were euthanized by isoflurane inhalation before cervical dislocation.”

And in line 504-507

“Mice sacrificed at the onset of diabetes prior to detection of symptoms to prevent suffering and were perfused with collagenase (Vitacyte, CIzyme catalog #005-1030) and dissected to remove the pancreas for further processing.”

**3. Please note that PLOS ONE has specific guidelines on code sharing for submissions in which author-generated code underpins the findings in the manuscript. In these cases, we expect all author-generated code to be made available without restrictions upon publication of the work. Please review our guidelines at** [**https://journals.plos.org/plosone/s/materials-and-software-sharing#loc-sharing-code**](https://urldefense.com/v3/__https://journals.plos.org/plosone/s/materials-and-software-sharing*loc-sharing-code__;Iw!!DZ3fjg!-Wser9g61HL-T-rlfhdktLIyStMmWJl23m2BaQ2rbYwpCNtklsOcY--FUd6CWnjxZCuGiq1qPx1RSPBK9A$) **and ensure that your code is shared in a way that follows best practice and facilitates reproducibility and reuse.**

**Authors’ response:** All code is reproducible and has been made available for the public at <https://github.com/szimmerman92/NOD_t1d_islets_pbmc_single_cell_tcr>

Data is also publicly available and can be accessed through the NCBI GEO database with accession code **GSE200695.**

**4. Thank you for stating in your Funding Statement:
This study was supported by grants from the Beatson Foundation (Grants ID 2800013). The salary of the author MZI was supported by Lundbeck Foundation, Copenhagen, Denmark (Grants ID R288-2018-1123).**

**Please provide an amended statement that declares *all* the funding or sources of support (whether external or internal to your organization) received during this study, as detailed online in our guide for authors at** [**http://journals.plos.org/plosone/s/submit-now**](https://urldefense.com/v3/__http://journals.plos.org/plosone/s/submit-now__;!!DZ3fjg!-Wser9g61HL-T-rlfhdktLIyStMmWJl23m2BaQ2rbYwpCNtklsOcY--FUd6CWnjxZCuGiq1qPx2kap463w$)**. Please also include the statement “There was no additional external funding received for this study.” in your updated Funding Statement.
Please include your amended Funding Statement within your cover letter. We will change the online submission form on your behalf.**

**Authors’ response:** We have amended the funding statement in the manuscript to include:

“This study was supported by grants from the Beatson Foundation (Grants ID 2800013). The salary of the author MZI was supported by Lundbeck Foundation, Copenhagen, Denmark (Grants ID R288-2018-1123). This includes all of the funding sources that supported this grant and there were no additional external funding sources.”

**5. Please note that your Data Availability Statement is currently missing the direct link to access each database. If your manuscript is accepted for publication, you will be asked to provide these details on a very short timeline. We therefore suggest that you provide this information now, though we will not hold up the peer review process if you are unable.**

**Authors’ response:** We have amended our data availability statement to include the direct link to the data. Please see line 631-636.

**6. When completing the data availability statement of the submission form, you indicated that you will make your data available on acceptance. We strongly recommend all authors decide on a data sharing plan before acceptance, as the process can be lengthy and hold up publication timelines. Please note that, though access restrictions are acceptable now, your entire data will need to be made freely accessible if your manuscript is accepted for publication. This policy applies to all data except where public deposition would breach compliance with the protocol approved by your research ethics board. If you are unable to adhere to our open data policy, please kindly revise your statement to explain your reasoning and we will seek the editor's input on an exemption. Please be assured that, once you have provided your new statement, the assessment of your exemption will not hold up the peer review process.**

**Authors’ response:** We have released the data in anticipation of the manuscript being accepted for publication at PLOSONE.

**7. We note that you have included the phrase “data not shown” in your manuscript. Unfortunately, this does not meet our data sharing requirements. PLOS does not permit references to inaccessible data. We require that authors provide all relevant data within the paper, Supporting Information files, or in an acceptable, public repository. Please add a citation to support this phrase or upload the data that corresponds with these findings to a stable repository (such as Figshare or Dryad) and provide and URLs, DOIs, or accession numbers that may be used to access these data. Or, if the data are not a core part of the research being presented in your study, we ask that you remove the phrase that refers to these data.**

**Authors’ response:** We have modified the figure legend for figure 1 to remove this phrase as it was not necessary in the first place.

**Editor comments:**

Thank you for providing your additional comments in lieu of a second reviewer. We believe that these comments have allowed us to strengthen the manuscript and discover new insights about our hypothesis. Below we will answer the concerns that you have mentioned.

**1) In the discussion section (line 372), it should be clarified that the lpr mutation in the lupus-prone MRL genetic background (MRL/lpr) or in the normal B6 genetic background (B6/lpr) does not result from a null mutation in the fasL gene as indicated in the discussion. Instead, it arises due to the insertion of the retrotransposon ETn in the second intron of the fas gene, leading to transcriptional repression. Therefore, lpr mice, like NOD mice, possess functional copies of fasL genes. Additionally, DN T cells and CD8+ T cells from MRL/lpr or B6/lpr mice exhibit elevated expression of functional FasL on the plasma membrane. Furthermore, to the best of my knowledge, the current understanding is that pathogenic DN T cells in lpr mice are mainly generated in the periphery as a result of dysregulated homeostasis of effector/memory T cells.**

**Authors’ response:** This is a very pertinent comment for the manuscript as the DN population over-representation, which has been identified before in NOD mice through wet lab assays, was the most surprising result found in our paper. As the editor has pointed out, this phenomenon was previously highly researched in the MRL/lpr genetic background. We concur that we have inaccurately characterized the genetic defect in this background and have taken the steps necessary to rectify this. We have changed the discussion of the manuscript to read. Please see line 414-418.

“~~Studies in Lpr mice suggest that these FasL null mutants cannot apoptose DN cells that develop in the thymus or periphery. NOD mice have a functional copy of FasL but the NOD genetic idd loci do contain genes involved in T cell signaling like Ptpn22 and Ctla4.~~

Studies in the MRL-lpr mice which contain a spontaneous mutation in the Fas receptor have connected lymphoproliferation of DN T cells to a reduced apoptotic signal of negatively selected T cells in the thymus (Jeltsch-David and Muller 2014). The genetic similarities between these mouse models are limited as Fas have not been identified as one of the IDD loci and diabetic onset in NOD mice is likely polygenic (Chen et al. 2018). ”

We hope this rectifies the mischaracterization of the similarities between the lpr mouse model and the NOD mouse model. In the next response we explained the similarities with further validation experiments.

**2) The authors should provide a detailed explanation of their methodology to demonstrate that the DN cells observed in NOD mice arise from their own proliferation, as stated line 403 (conclusion), rather than from the conversion of CD8+ cells into DN cells. Indeed, DN T cells could originate from CD8+ T cells, as shown in various lupus mouse models where chronic activation leads CD8+ T cells to downregulate their CD8 molecules. Therefore, have you quantified CD8 surface levels (MFI) in NOD CD8+ T cells using flow cytometry? Additionally, do DN T cells in NOD mice overexpress the phosphatase B220 (CD45RABC) along with Fas ligand, similar to DN T cells in lupus mouse models?**

**Authors’ response:** We also thank the editor for these comments. As they point out, a significant amount of research has gone into the double negatives in both the lpr and NOD backgrounds and there are other possible populations of double negatives that could make up the majority of these cells. We attempted to investigate these populations by running a new flow cytometry on 24-week-old NOD mice using pooled diabetic and non-diabetic splenocytes (because non-diabetic may be pre-diabetic in this instance it would not be significant to separate them). The flow gating strategy is shown below and is part of a new supplementary figure (Supplementary Figure 5):

The most important findings were that there was a majority of the DN T cell population that were B220^+^. However, they were also largely FasL^-^, and NK1.1^-^.

This validates that NOD mouse double negatives are not NKT cells. The discovery of B220 is in line with the previous reports in lpr mice and has never been reported before in the NOD background. However, in contrast with the lpr findings, the majority of NOD DN T cells are FasL negative which could indicate a unique pathway to the development of DN that is different from the lpr mouse which is suspected to be caused by insufficient apoptotic signaling of negatively selected cells in the thymus. To address the question about the potential CD8+ origin of the NOD DN T cells, we repeated the flow cytometry in thymocytes extracted from the same mice. We do observe a slight increase in the number of B220+ CD8 T cells which could be the progenitor cell type of the B220+ DN T cell.

However, there is also evidence that would indicate otherwise, for instance, we see in Fig 3b-d that total CD8 numbers increase steadily over time while CD4 T cell populations fluctuate peripherally in line with DN T cells.

We also see as much similarity between DN T cells expression and CD4 populations. From the various results we hypothesize that the DN T cell population can come from either SP source. We agree that the origin of this cell type is very important to how they may affect disease onset. The experiments needed to validate their source are too numerous for the scope of this manuscript and will be something that we will continue to investigate in the future.

It is curious that we found reduced TCR beta surface expression in the DN T cells, where other sources find that these populations are TCR alpha beta positive (Hamad et al., 2003). We did confirm that these are not TCRgamma delta cells and reduced surface TCR beta is consistent with an observed decrease in TCR expression overall in the DN T cells. Perhaps it is because we are observing older mice and DN lose TCR beta over time as part of some exhaustion mechanism making them instead TCRBeta_low_.

This new data represents validation of the cell types that were originally hypothesized and connection to previously published studies that can allude to the possible origins and function of these cells.

To incorporate the new data, we have changed the methodology of the flow cytometry. Please see line 612-625.

“For flow cytometry analysis, T-cell enriched samples from the blood of 5 diabetic and 5 non-diabetic NOD mice at 6,14,20 and 24 weeks of age were stained using labeled primary antibodies against CD4 (GK1.5, Thermofisher #12-0041-82), CD3e (17A2, Thermofisher #48-0032-82), CD8a (53-6.7, Thermofisher #56-0081-82), and CD8b (H35-17.2, Thermofisher #11-0083-82), NK1.1 (PK136, Thermofisher #407-5941-82), TCRB (H57-597, BioLegend #109225), TCRgd (), B220 (RA3-6B2, BioLegend #103212), FasL (MFL3, Thermofisher #63-5911-82), Fas (15A7, Thermofisher #46-0951-82), CD19 (1D3, Thermofisher #M004T02Y03-A), and TCRgd (GL-3, Thermofisher #25-5711-82).”

We have also changed the results section 3 to reflect this new information and also to make results more easy to understand. Please see line 171-192.

“First, we stained pooled splenocytes and thymocytes from 24 week non-diabetic and diabetic NOD mice with markers for CD3, CD4, CD8b, FasL, B220, TCRB, and NK1.1 (**Supplementary Figure 5a**). We were surprised to see that CD3+ DN splenocytes made up 25% of CD3+ T cell populations in the spleen (**Supplementary Figure 5b**). Consistent with reports from MRL/lpr mice [(Asano et al. 1988)](https://sciwheel.com/work/citation?ids=17187613&pre=&suf=&sa=0), >30% of the DN were B220^+^, however, they did not express more FasL than CD4+ or CD8+ T cells. As expected, none of these DN T cells bore the NK1.1 marker for NKT cells, however we did observe reduced MFI for TCRβ on the cell surface. We have confirmed through additional analysis that this did not correlate with an increase in gamma delta TCR abundance (**Supplementary Figure 5c,d**). Thymic populations had relatively fewer CD3+ DN T cells (<20%), however these populations were still B220^+^ and TCRβ_low_ but had greater expression of FasL and NK1.1 than CD4+ and CD8+ populations (**Supplementary Figure 5 e,f**).

We were interested to see how populations of DN T cells changed over time in diabetic mice so we conducted flow cytometry on paired blood samples from diabetic and non-diabetic mice used in single cell analysis at 4 different time points (**Figure 3a**). We have determined that the DN T cell compartment in the blood is negatively correlated with CD4+ populations but not correlated with CD8+ populations that increase steadily over a 24 week period (**Figure 3b**). Diabetic condition results in a shift from mostly CD4+ T cells to mostly DN T cells by week 24, whereas CD4+ T cells remain slightly more abundant throughout the same time frame in non-diabetic mice ~~We find that in diabetic mice, CD4+ T cells make up a significantly higher proportion of T cells at 14 weeks of age than DN T cells and while this levels out by week 24 in the non-diabetic mice, the DN proportion flips and becomes significantly higher in the diabetic mouse~~ (**Figure 3c**). “

And have added the following sentences to the discussion. Please see line 418-429.

“However, we have confirmed similarities between lpr DN and NOD DN, including increased amounts of B220 expression, even though there is seemingly no change in expression of the CD45 gene. These similarities may hint at a common development pathway, however many of our results such as single cell trajectories and cross DN-CD4 clonal lineages would suggest peripheral development of DN T cells not seen in lpr mice.”

We have added a new supplementary figure, Supplementary Figure 5, to support the new findings.

**Supplementary Figure 5.** Validation of DN populations were performed using flow cytometry. (a) Splenocytes were pooled from 24 week old diabetic and non-diabetic NOD mice and then stained for T cell specific markers. (b) Boxplots show populations of CD3+ splenocytes of DN, CD4+ and CD8+ T cells. (c) Histogram of TCRB MFI shows lower protein abundance of TCR on the surface of DN T cells than CD4+ T cells. (d) Splenocytes largely are not expressers of gamma delta TCR. (e) Overall and (f) marker populations of CD3+ T cells in thymocytes.

Hamad, Abdel Rahim A., et al. "B220+ double-negative T cells suppress polyclonal T cell activation by a Fas-independent mechanism that involves inhibition of IL-2 production." *The Journal of Immunology* 171.5 (2003): 2421-2426.

**Reviewer #1 comments:**

**1) In the Materials and Methods section "Blood glucose measurement and diabetes diagnosis" (line 433), I suggest including a reference for the parameters used to diagnose diabetic mice (two consecutive glucose readings of ≥250 mg/dl considered diabetic).**

**Authors’ response:** We appreciate the reviewer for this advice. We have revised this section in the methodology and added references (please see line 491-492). The criteria for diagnosis of blood glucose in the NOD mouse can vary between studies. Diabetogenesis in the NOD mouse is caused by islet beta cell destruction >80% resulting in a marked decrease in insulin production. This cannot be directly observed without sacrifice and microscopic observation of the pancreas, and so blood glucose is an analogue to this autoimmune damage *in-vivo*. The baseline for diagnosis can be difficult to decide on because glucose readings can spike past 250 mg/dL and then return to resting levels, which is why we required two consecutive readings taken within a two week period. At which point mice were sacrificed and then pooled by a week of diabetic onset. The 250 mg/dL measurement has been used in our lab for a long time after previous prospectus study (Vatanen et al., 2016) and is also used by other labs to great success as demonstrated in (Clayton et al., 2015).

Vatanen, Tommi, et al. "Variation in microbiome LPS immunogenicity contributes to autoimmunity in humans." *Cell* 165.4 (2016): 842-853.

Mathews, Clayton E., et al. "Acute versus progressive onset of diabetes in NOD mice: potential implications for therapeutic interventions in type 1 diabetes." *Diabetes* 64.11 (2015): 3885-3890.

**2) Figure 1D, results: Is it possible to evaluate the frequency of T cells in the islets of non-diabetic mice using flow cytometry?**

**Authors’ response:** We thank the reviewer for their question; However, it is unclear exactly what aspect they are referring to. If the question is in regard to Figure 1D, the cell counts in this figure were taken by automated cell count of all CD3 cells purified by magnetic beads that were pooled then used in single cell RNA sequencing.

Islets were isolated through previously described methods before CD3+ T cell purification and must be done at time of collection. Because T cell counts would have been too low for single cell input even after pooling, non-diabetic islets were not isolated. However, it is previously known the expected count of T cells in non-diabetic islets (Magnuson et al., 2015). As table S1 in this paper shows, the number of CD45+ lymphocytes infiltrates in non-insulitic (non-diabetic equivalent) NOD mice into the pancreas is less than 0.03% of normal NOD mice at 8 weeks of age. The protocol at the time of sequencing (2018, Chromium v1) from 10X genomics recommended at least 20,000 cells input so we would not have had enough input for reasonable cell recovery at that time even after pooling.

Non-diabetic mice were also not comparable to diabetic mice due to extreme differences in age (>15 weeks in the worst cases). Comparison of T cell expression between pancreatic islets of diabetic and non-diabetic mice will be more viable in future publications where we will be performing spatial transcriptomics. This work is currently ongoing.

Magnuson, Angela M., et al. "Population dynamics of islet-infiltrating cells in autoimmune diabetes." *Proceedings of the National Academy of Sciences* 112.5 (2015): 1511-1516.

**3) Considering the complementary determining region (CDR3) of autoreactive T cell clones, it could be interesting to determine the antigen fragments potentially recognized using the BLAST tool. This could reinforce that, in particular, Trav16n, Trbv29, and Trav8d-2 exhibit CDR3 regions that may potentially recognize insulin peptides.**

**Authors’ response:** We thank the reviewer for commenting on this aspect of the study. As we stated, we did look for exact matches of CDR3 that already exist in databases like IEdb and VDJDB but could only find two matches in the expanded clones. We were particularly focused on these clones which contain greater than 10 clones in the dataset, because the pathogenic specificity would likely be higher. However, to fully answer the reviewer’s question we re-aligned the CDR3 sequences in our dataset from all of our cells to the VDJdb database and found positive hits for exact matches of 4,166 cells (added to new supplementary table S16). There were no H2g7 restricted epitopes among the matches and most (>99%) were MHC class I restricted and specific to viral targets mostly influenza A and MCMV. There were a few mouse targeted peptides, however none were insulin specific, with the most abundant target being Myelin Basic Protein, which may be cross presented on H2g^7^ as NOD mice are susceptible to Experimental Autoimmune Encephalitis. We observe no cluster specificity for any given epitope target, however curiously Influenza NP was the only epitope which had epitope specific T cells at higher normalized rates invading in the pancreas. We blasted the epitope sequence to find 100% homology to mouse Keratin 222. While this is expressed in the pancreas, there is not a huge amount of evidence for beta cell specificity. At the very least we confirmed that these epitope targeted T cells were present in every single mouse from our SPF facility, so it is unlikely that these are the result of an unknown viral infection. We note that a recent study in Japan found infection by Influenza A to be associated with an increase in T1D incidence (Noshioka et al., 2021).

These databases hold over a million records of experimentally validated TCR specificity experiments and document specific CDR3 regions, MHC, and epitope. We foresee one of two possible reasons for the lack of diabetic target specificity. One is incorrect annotation of the CDR3 region in our dataset, as our analysis used early versions of the 10X cell ranger pipeline that may have failed to get the correct exact CDR3 sequence. Two is that there are few NOD specific records at less than 400 total entries for the NOD mouse background. The problem with blast or Levenshtein search of CDR3 regions, is that it can consider non-exact matches (gaps, mismatch, insertions) of the CDR3 region which can result in wildly different specificity and is not directly relatable to experimentally validated data.

However, the reviewer’s comment made us realize we failed to look more broadly at our expression data for TCR genes, which we show have specificity for islet invasion and may have cell type specificity as well. We rectified this by observing gene specific expression of all TCR genes including gamma delta TCR. We do find an interestingly high amount of gamma delta TCR expression in the exhausted/overactive CD8+ and DN TC1-like cells. Interestingly, the DN population has higher specificity for Trav15d-1 and both have higher expression of Trav16n which was identified to predict for islet infiltration. Trbv29 also seems to be more specific for CD8+ T cells. Effector DN T cells have relatively higher expression of Trav15n and Trav15d than their CD4 equivalent. Trav15n is focused in the pre-exhausted/overactivated T effector cluster and is specific to the Influenza A PA (SSLENFRAYV) and PB1 (LSLRNPILV) antigens in VDJDB while Trav15d has specificity to Plasmodium Berghei GAP50 (SQLLNAKYL).

DN T cells seem to undergo reduced expression of TCRab genes without an equivalent rise in TCRgd, which we have confirmed by observing lower TCRab MFI in the splenocytes. Perhaps this is an underlying mechanism of exhaustion or overactivation which results in reduced TCR presentation as other studies suggest (Valitutti et al., 1997).

To incorporate these new changes, we have modified the results section 4 to include:

“To investigate whether identified T cell clonal populations were targeting previously known epitopes associated with diabetic progression we searched for exact CDR3 matches to the VDJdb (cite), a database of experimentally validated TCR-pMHC interactions. We only found two hits of the expanded (>10) clones but among all clones we found 4,166 matching CDR3 sequences in the VDJdb (Supplementary File 19). Of the known epitopes sources to the matching CDR3 sequences, the most common was to Influenza A PA (686 hits) and PB1 (392 hits) with the second highest target being Murine Cytomegalovirus (MCMV) M45 (724 hits) along with recognition of epitopes from Lymphocytic Choriomeningitis Virus (LCMV), Plasmodium Berghei, and Respiratory Syncytial Virus (RSV). There were much fewer self-targeted antigens with the majority specific to Myelin Basic Protein (MBP) and Protein Arginine Deaminase 4 (PADI4), we did not observe any specific to known insulin specific epitopes. While the majority of the antigen specific T cells were evenly distributed among tissues and clusters, we observed Influenza NP (ASNENMETM) targeting T cells had higher relative numbers in the pancreatic islets (Supplementary Figure 9e-f).”

We have then also modified results section 5 to include:

“We looked at specific expression of TCR genes among clusters to see if specific TCR gene expression related to invasion and diabetes (Supplementary Figure 10). We do find an interestingly high amount of gamma delta TCR expression in the exhausted/overactive CD8+ and DN TC1-like cells, although they remain <5% of all T cells. Interestingly, the DN population has higher specificity for Trav15d-1 and both have higher expression of Trav16n which was identified to predict for islet infiltration. Trbv29 also seems to be more specific for CD8+ T cells. Effector DN T cells have relatively higher expression of Trav15n and Trav15d than their CD4 equivalent (Supplementary Figure 10d). Based on entries in the VDJdb (cite), Trav15n, which is focused to the IL7R- T effector cluster and is specific to the Influenza A PA (SSLENFRAYV) and PB1 (LSLRNPILV) antigens in VDJDB while Trav15d expressed in IL7R+ DN T effector cells has specificity to Plasmodium Berghei GAP50 (SQLLNAKYL).”

Additionally, we have added the following to the discussion:

“We did not identify any insulin specific peptides as targets of our dataset TCR, possibly due to a combination of few NOD experiments in VDJdb and poor CDR3 recognition in the TCR sequencing. We did find some targets which could have further impact on diabetic research, most notably a higher relative pancreatic infiltration of Influenza A NP targeted T cells, which previous studies have linked viral infection to greater incidence of T1D (Nishioka et al. 2021). BLAST search of the epitope to self-antigenic proteins in mice resulted in a 100% match (NENMET) to Keratin 222 (KRT222).”

We have then modified Supplementary Figure 8 (Now as Supplementary Figure 9) to be:

**Supplementary Figure 9.** Lineage tracing and expression of infiltrating T cell clones. (a) Per TCR monocle trajectories plotted for each cell in the diabetic (left) and non-diabetic (right) mice. Each line represents the trajectory of one TCR clone across UMAP positions. (b) Volcano plot showing differential expression of genes in the infiltrating vs non-infiltrating T cells in diabetic mice with positive log fold change in the infiltrating T cells. Significant log fold change values > 1 and adjusted P-values < 0.01 are colored red and top 25 significant genes are labeled. (c) Violin and box plot showing average expression of Ifng between tissue matched (infiltrating) and non-matched T cells. (d) Likelihood estimation for bootstrapped pair transitions within clones. Each clone was randomly sampled for pairs of cells 100 times and the likelihood of each transition was calculated based on the proportion of two different cell types being chosen. The results were plotted as a graph with the nodes representing each cell cluster. The thickness of each bar represents the average percent likelihood of a transition occurring across clones with more than 3 cells. Red colored lines represent transitions occurring within the same compartment (i.e., CD4 to CD4) while blue colored lines represent transitions occurring between compartments (i.e., DN to CD4). The direction of transition cannot be inferred however the nodes are organized from bottom to top in order of ascending T cell maturation. (e) Epitope targets were predicted from the VDJdb database and cells with exact matches were plotted on the cell UMAP and colored by target epitope. (f) Normalized cell numbers were plotted based on targeted epitope by tissue.

We have also Added a new Supplementary Figure 10 to include:

**Supplementary Figure 10.** Expression of TCR genes by cluster. Dot plot showing cell percentage and average expression in each cluster for tcr alpha (a), beta (b), and gamma/delta (c). (d-f) Psuedobulk expression plots showing average expression of tcr gene expression by cluster.

Valitutti, Salvatore, et al. "Degradation of T cell receptor (TCR)–CD3-ζ complexes after antigenic stimulation." *The Journal of experimental medicine* 185.10 (1997): 1859-1864.

Nishioka, Yuichi, et al. "Association between influenza and the incidence rate of new‐onset type 1 diabetes in Japan." *Journal of Diabetes Investigation* 12.10 (2021): 1797-1804.

With regards,

**MICHAEL ROBBEN**
*Assistant Professor*
 
College of Agricultural, Consumer and Environmental Sciences (ACES)
Department of Animal Sciences

1201 W Gregory Dr

ERML 227A

Urbana, IL 61801

[Robben@illinois.edu](mailto:Robben@illinois.edu)

217-244-4758

[Illinois Laboratory for Animal Machine Learning and Bioinformatics](http://robbenlab.org/)

<http://illinois.edu/>

*Under the Illinois Freedom of Information Act any written communication to or from university employees regarding university business is a public record and may be subject to public disclosure*
