# Supplementary material for: Single-cell RNA-seq reveals disease-specific CD8+ T cell clonal expansion and a high frequency of transcriptionally distinct double-negative T cells in diabetic NOD mice
Source: PLoS One. 2025 Mar 19;20(3):e0317987. doi: 10.1371/journal.pone.0317987 (PMC11922263; doi:10.1371/journal.pone.0317987)
Supplement: Attachment — Submitted filename: Corrected_NOD_PlosONE_author_response_1_6_25.docx [file pone.0317987.s013.docx]

Dear PLOSONE editors and reviewers,

The authors would like to thank the editor and reviewers for their consideration of this manuscript and for their help in making the modifications necessary for publication into PLOSONE. The authors have reviewed the suggested changes and have taken steps to fix and modify the manuscript to bring it more in line with the quality of PLOS ONE. The authors have listed and detailed the changes made below, addressing each comment individually. Please let us know if any changes are not satisfactory or if anything else needs to be adjusted to address any concerns.

Additional Editor Comments:

Please review your reference list to ensure that it is complete and correct. If you have cited papers that have been retracted, please include the rationale for doing so in the manuscript text, or remove these references and replace them with relevant current references. Any changes to the reference list should be mentioned in the rebuttal letter that accompanies your revised manuscript. If you need to cite a retracted article, indicate the article’s retracted status in the References list and also include a citation and full reference for the retraction notice.

The authors have reviewed their reference list and have concluded that no retracted articles are included among citations. If the authors are incorrect, please let us know which articles are retracted and we can take the necessary steps to either remove or justify the use of such an article in this manuscript.

- However, some minor points persist in the text, such as the inconsistent spelling of "pseudo," which is incorrectly written as "psuedo" at least six times.

Thank you for pointing this out before it went to publication. We have taken the opportunity to fix all incorrect appearances of the word “pseudo”.

- Furthermore, I recommend that the authors include the FSC-A vs. SSC-A dot plot in their gating strategy (Supplementary Figure 5) to demonstrate that only single cells are included in the flow cytometry analysis, while doublets or multiplets are excluded.

This was an unnecessary omission and we have corrected this by amending supplementary Figure 5.

The figure was amended to add the FSC-H by FSC-A and all graphs were amended to label FSC and SSC by Height or Area. The figure legend for supplementary figure 5 has not been changed and is in the current version of the revised manuscript.

- I found the statements regarding Fas-deficient lupus mice in the Discussion section on page 20 to be ambiguous in the first instance and inaccurate in the second: “Studies in the MRL-lpr mice ….. have connected lymphoproliferation of DN T cells to a reduced apoptotic signal of negatively selected T cells in the thymus [50], as well as “ …….would suggest peripheral development of DN T cells not seen in lpr mice. There is substantial evidence indicating that peripheral DN T cells are generated in the periphery of lpr mice and patients with autoimmune lymphoproliferative syndrome (ALPS), rather than representing primitive αβ T cells that escape late-stage thymocyte development before migrating to peripheral tissues. Specifically, multiple reports have shown that B220^+^ DN T cells originate from CD8^+^ T cells at the effector/memory stage following repeated TCR engagement in Fas-deficient lupus mice. Similar findings have also been reported in human studies.

Thank you for your input on this matter. We are less familiar with the MRL/lpr mouse model and thus made an egregious error after not looking at the existing literature extensively enough. We have amended the above cited sections to better reflect the known developmental pathway of T cells in the MRL/lpr mouse model. The changes are copied below.

Studies in the MRL-*lpr* mice which contain a spontaneous mutation in the Fas receptor have connected lymphoproliferation of DN T cells to a reduced ability to remove defective thymic or peripheral T cells. The ability for the lpr and gld mutations to protect NOD mice from T1D correlated with our finding that diabetic mice undergo greater expansion of DN T cells, perhaps as protective mechanism of peripheral tolerance.

We have confirmed similarities between *lpr* DN and NOD DN T cells, including increased amounts of B220 expression, even though there is seemingly no change in expression of the CD45 gene. These similarities may hint at a common development pathway, including likely peripheral development according to the single cell trajectory and clonal lineage tracing.

- To avoid any confusion for the readers, I recommend adding "SP" when referring to the CD4+ and CD8+ phenotype, for instance, CD4/DN and CD4+/CD8+ could be written CD4+ SP/DN and CD4+/CD8+ SP T cells.

You are correct. We see now how mentioning multiple subtypes together could confuse the reader. To make it unambiguous, whenever a subtype is mentioned in combination with others, we have added “SP” to denote that a specific subtype is not DP nor DN.

- Additionally, when mentioning the MRL/lpr mouse strain, I suggest including the term "lupus-prone" (e.g., lupus-prone MRL/lpr mice) for those who may not be familiar with this mouse model of systemic lupus erythematosus (page 9).

This is a good suggestion; we have made the edits accordingly.

Studies in the lupus prone MRL-*lpr* mice which contain a spontaneous mutation in the Fas receptor have connected lymphoproliferation of DN T cells to a reduced ability to remove defective thymic or peripheral T cells

Additionally, the authors have attached the revised manuscript with a track changes copy to this submission. Please let us know if there are any other changes that would need to be made before publication.

With regards,

**MICHAEL ROBBEN**
*Assistant Professor*
 
College of Agricultural, Consumer and Environmental Sciences (ACES)
Department of Animal Sciences

1201 W Gregory Dr

ERML 227A

Urbana, IL 61801

[Robben@illinois.edu](mailto:Robben@illinois.edu)

217-244-4758

[Illinois Laboratory for Animal Machine Learning and Bioinformatics](http://robbenlab.org/)

<http://illinois.edu/>

*Under the Illinois Freedom of Information Act any written communication to or from university employees regarding university business is a public record and may be subject to public disclosure.*
